# Sex differences in dengue-related neurological complications: insights from the 2023 Taiwan outbreak

**DOI:** 10.1093/braincomms/fcag258

**Published:** 2026-07-07

**Authors:** Wen Zhen Lin, Poh Shiow Yeh, Ling Shan Hsueh, Hung Jen Tang, Che Chun Yeh, Nan Yao Lee, Sheng Hsiang Lin, Yuan Ting Sun

**Affiliations:** Department of Neurology, An Nan Hospital, China Medical University, Tainan 709, Taiwan; Department of Neurology, Chi Mei Medical Center, Tainan 710, Taiwan; Department of Internal Medicine, National Cheng Kung University Hospital, College of Medicine, National Cheng Kung University, Tainan 704, Taiwan; Department of Infectious Diseases, Chi Mei Medical Center, Tainan 710, Taiwan; Department of Neurology, National Cheng Kung University Hospital, College of Medicine, National Cheng Kung University, Tainan 704, Taiwan; Division of Neurology, Department of Internal Medicine, National Cheng Kung University Hospital, Yunlin 640, Taiwan; Department of Internal Medicine, National Cheng Kung University Hospital, College of Medicine, National Cheng Kung University, Tainan 704, Taiwan; Institute of Clinical Medicine, College of Medicine, National Cheng Kung University, Tainan 704, Taiwan; Biostatistics Consulting Center, National Cheng Kung University Hospital, College of Medicine, National Cheng Kung University, Tainan 704, Taiwan; Department of Public Health, College of Medicine, National Cheng Kung University, Tainan 704, Taiwan; Department of Neurology, National Cheng Kung University Hospital, College of Medicine, National Cheng Kung University, Tainan 704, Taiwan; Department of Medical Genomics, National Cheng Kung University Hospital, College of Medicine, National Cheng Kung University, Tainan 704, Taiwan

**Keywords:** dengue, neurological manifestation

## Abstract

Dengue is a prevalent vector-borne disease that threatens nearly half of the world’s population. Although not traditionally classified as neurotropic, dengue can lead to a wide range of neurological manifestations beyond the conventional spectrum of disease severity. This study investigated neurological manifestations during the 2023 dengue outbreak in Tainan, Taiwan, with a focus on the observed male predominance in symptomatic and severe cases. In 2023, the national infectious disease reporting system recorded 21 513 dengue cases in Tainan, of whom 53% were male. Among these, 768 patients (56.4% male; 10.2% paediatric) required hospitalization in tertiary medical centres. Neurological manifestations were observed in 12.0% of hospitalized patients, corresponding to 0.43% of all reported dengue cases in the Tainan area. Among hospitalized patients, neurological manifestations included encephalitis (0.4%), myelitis (0.1%), myositis (7.8%), encephalopathy (2.0%), intracranial haemorrhage (1.0%), ischaemic stroke (0.9%), myasthenia gravis (0.4%), seizures (1.0%), Guillain–Barré syndrome (0.8%), and opsoclonus–myoclonus syndrome (0.1%). Myositis was the most common neurological manifestation, occurring in 26.9% of paediatric and 5.7% of adult patients. Risk factors for neurological complications included male sex, coexisting chronic kidney disease, and neurodegenerative disorders. Male patients showed a higher likelihood of symptomatic dengue infection in the overall population (odds ratio 1.15, 95% confidence interval 1.12–1.18), as well as increased risks of severe disease, hospitalization in tertiary medical centres (crude odds ratio 1.25, 95% confidence interval 1.06–1.46), and neurological complications (adjusted odds ratio 2.37, 95% confidence interval 1.32–4.26) among those older than 40 years. Although the overall case fatality rate in 2023 was 0.9 per 1000 cases, neurological complications were relatively frequent among hospitalized patients. These findings highlight the need for heightened clinical awareness of dengue-associated neurological complications, particularly among neurologists. The consistent male predominance across symptomatic infection, hospitalization, and neurological involvement suggests that sex may be an independent determinant of dengue severity and neurological risk, especially in older adults. Incorporating sex-specific risk stratification and comprehensive neurological assessment into dengue care may improve early detection and clinical management during future outbreaks.

## Teaser

Lin et al. report that during the 2023 dengue outbreak in Tainan, neurological complications occurred in 12% of hospitalized patients, with myositis as the most common manifestation. Male sex was independently associated with symptomatic infection, severe disease, and neurological complications, highlighting important sex differences in dengue-related neurological involvement.

## Graphical Abstract

**Graphical Abstract:**
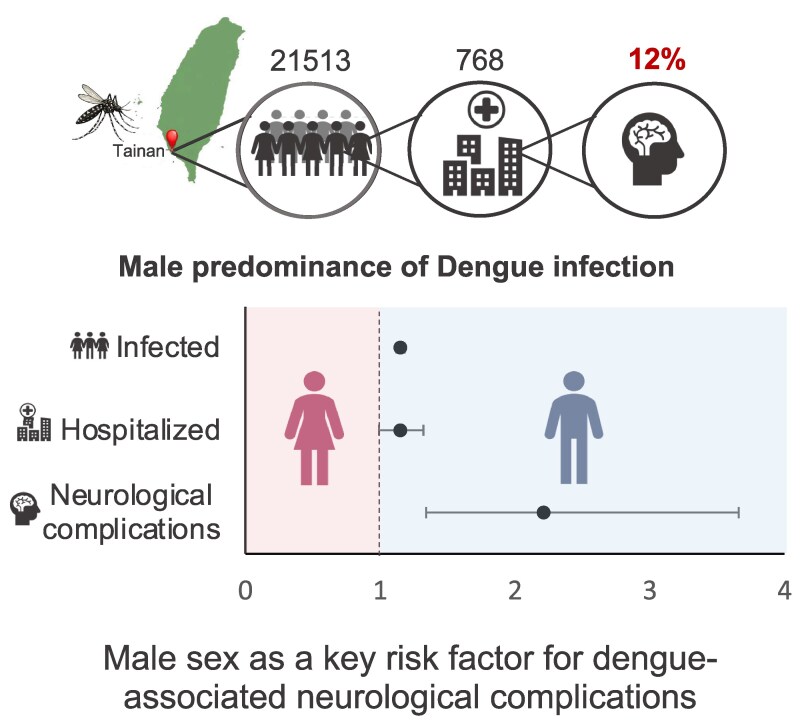

## Introduction

Dengue is one of the most prevalent vector-borne flaviviral infections worldwide, with four distinct serotypes (I–IV).^1,2^ The endemic region spans over 100 countries, placing nearly half of the global population at risk. Annually, an estimated 100–400 million dengue infections occur.^3,4^

The clinical manifestations of dengue infection vary widely, ranging from asymptomatic cases to severe and life-threatening shock syndrome.^5,6^ Although traditionally not considered a neurotropic virus, reports of neurological complications associated with dengue have been increasing. These complications can be categorized into three hypothetical mechanisms: first, direct invasion of the central nervous system (CNS), causing encephalitis, meningitis, or myelitis; second, systemic complications leading to conditions such as encephalopathy or intracranial haemorrhage (ICH); and third, parainfectious immune-mediated responses. The latter includes acute-phase cytokine-mediated conditions like myositis, as well as delayed immune phenomena such as acute disseminated encephalomyelitis (ADEM), Guillain-Barré syndrome (GBS), and opsoclonus-myoclonus syndrome.^7-14^

Although the World Health Organization (WHO) has established guidelines with warning signs to aid clinical decision-making, their specificity and prognostic relevance remain uncertain. The evolving dynamics of dengue and its associated neurological complications continue to challenge timely diagnosis and management, particularly during outbreaks.

Despite growing recognition of dengue-associated neurological disease, the influence of biological sex on these complications remains insufficiently explored. Previous epidemiological studies have documented a male predominance in symptomatic and severe dengue,^15-17^ yet it is unclear whether this disparity extends to neurological involvement. Understanding sex-specific susceptibility is clinically important, as immunological and hormonal differences between males and females may shape host responses to viral infection.

Leveraging comprehensive population- and hospital-based data from the 2023 dengue outbreak in Tainan, Taiwan, this study aims to characterize neurological manifestations of dengue and to determine whether sex influences their occurrence and distribution. These insights may improve risk stratification and early recognition of dengue-associated neurological disease.

## Materials and methods

### Study design and ethical approval

This retrospective observational study was conducted at National Cheng Kung University Hospital (NCKUH) and Chi Mei Hospital (CMH) in Tainan, Taiwan. The study protocol was approved by the respective Institutional Review Boards (IRB) (Approval Nos. B-ER-113-148 and 11304-019). The study was conducted in accordance with the principles of the Declaration of Helsinki. As this was a retrospective study using de-identified clinical data, the requirement for informed consent was waived by the Institutional Review Board.

The dataset comprised anonymized population-level and hospital-based data from NCKUH and CMH. Population data for individuals diagnosed with dengue viral infection were retrieved from the National Infectious Disease Reporting System (NIDRS) via the Tainan City Government Public Health Bureau, covering the period from January 1, 2023, to December 31, 2023. Hospital-based data from the same period were collected from NCKUH and CMH.

### Definitions

In Taiwan, clinicians at all levels of medical institutions are obligated to report cases of recent dengue viral infection to the NIDRS. Reporting criteria include typical clinical presentations and a positive serum NS1 antigen test conducted 24 h after the first fever peak. The Taiwan Centres for Disease Control (CDC) provides rapid serology test kits to local clinics, regional hospitals, and medical centres.

**Figure 1:**
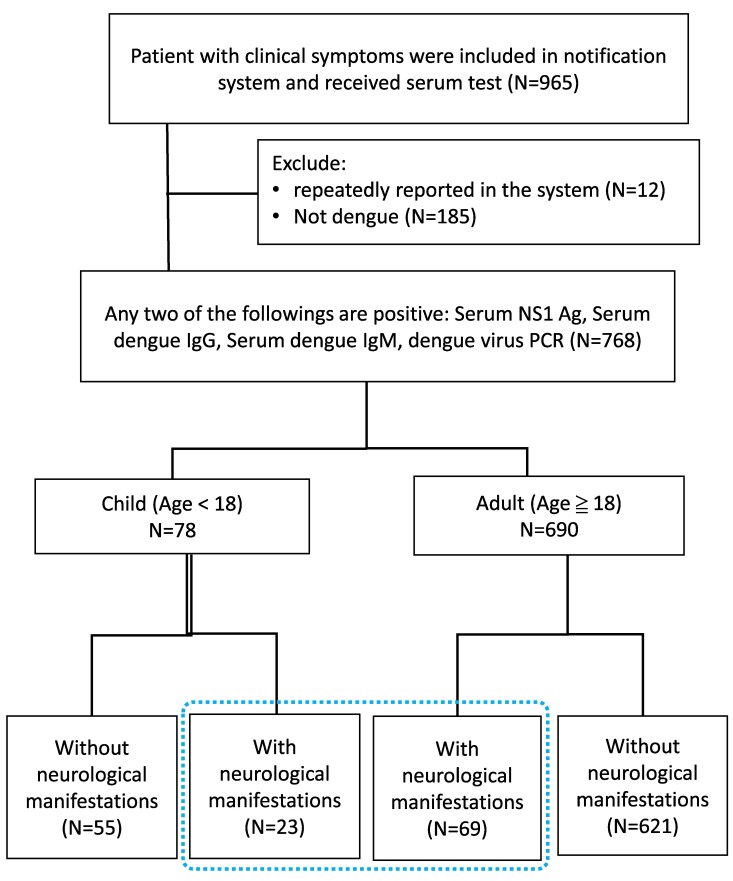
**Flowchart of patient enrollment.** Flowchart illustrating the identification and enrollment of hospitalized dengue patients during the 2023 Tainan outbreak. The dashed outline box highlights the subgroup of hospitalized patients who developed neurological manifestations.

Hospitalized patients diagnosed with dengue viral infection between January 1, 2023, and December 31, 2023, were identified using discharge ICD-10 codes A90, A91, A92, A93, and A94 from the electronic medical records (EMR) of NCKUH and CMH. Subjects were classified as paediatric or adult patients, with 18 years of age as the cutoff. A more stringent diagnosis of severe dengue syndrome was based on additional serological data, including dengue viral IgM, serial IgG levels, and reverse transcriptase polymerase chain reaction (RT-PCR) testing. Patients with positive dengue viral IgG but lacking antigen, PCR, or serial IgM data were excluded, as it was difficult to differentiate between recent infection and past events, such as the outbreak 7 years prior. Duplicate reports for the same patient from different medical institutions were resolved by removing one of the duplicates (Fig. 1).

Dengue virus typing was determined using RT-PCR on blood samples. The definition of dengue with warning signs followed the WHO criteria, which include persistent vomiting, abdominal pain, mucosal bleeding, lethargy, fluid accumulation, hepatomegaly, and an increasing haematocrit trend. Severe dengue infection was defined by the presence of life-threatening plasma leakage, severe bleeding, or organ impairment (involving the liver, kidneys, heart, or CNS).

The number of severe dengue cases was obtained from the medical centres and the Tainan City Public Health Bureau. During the outbreak, the Public Health Bureau required all medical institutions to report daily severe dengue case numbers to facilitate ICU bed allocation.

Data collected from the EMR included demographic information, lowest recorded body temperature, complete blood count (CBC), serum sodium and potassium levels, blood urea nitrogen (BUN), creatinine, creatine kinase (CK), AST, ALT, comorbidities, length of hospitalization, and clinical outcomes. The diagnosis of type 2 diabetes mellitus (T2DM) adhered to the criteria set by the American Diabetes Association,^18-21^ while chronic kidney disease (CKD) staging followed the guidelines of the National Kidney Foundation.^22^ Hepatitis was defined as AST and ALT levels exceeding twice the upper normal limit (UNL).

The neurological manifestations were categorized as infection-related or parainfectious events.^7,10,15,23^ Infection-related events are due to (1) the direct or cytokine-related effects of acute infection such as myositis (serum CK higher than 308 U/L in male patients and 192 U/L in female patients) and encephalitis with or without seizures; (2) the secondary complications, including traumatic ICH due to thrombocytopenia, metabolic encephalopathy; and (3) exacerbation of pre-existing neurological disorders during acute infection, such as myasthenia gravis (MG) crisis or acute exacerbation, and seizure recurrence in patients with epilepsy. Immune-related parainfectious events include GBS, myelitis, new-onset MG, and opsoclonus-myoclonus syndrome.

Definite dengue encephalitis was diagnosed based on encephalitic symptoms and the detection of the dengue virus in cerebrospinal fluid (CSF) and/or brain tissue through one or more of the following: NS1 antigen, RNA by PCR, virus isolation by culture, or viral antigen detection by immunohistochemistry.^11^ Japanese encephalitis virus (JEV) IgM serology testing was also performed to account for potential cross-reactivity between dengue and JEV. The key distinction between encephalitis and encephalopathy was the detection of dengue viral PCR or IgM in the cerebrospinal fluid (CSF), although both conditions share the hallmark feature of consciousness disturbance. The diagnosis of GBS followed a structured 10-step guideline.^24^ Dengue-associated stroke was defined as a stroke occurring within 2 months of dengue infection.^25^

### Comorbidities

Comorbidities in adult patients were assessed using the Charlson Comorbidity Index (CCI) score. Age was analyzed separately to account for the varying prevalence of different neurological syndromes and disorders across age groups.

### Statistical analysis

Statistical analyses were conducted using Prism (version 6; GraphPad Software, La Jolla, CA, USA). Unpaired Student's *t*-test, Mann–Whitney U-test, Kruskal–Wallis test, Chi-square test and Fisher's exact test were used according to data type, group number, and case number. Normality tests were conducted for continuous data before comparisons. Significance was set at *P* < 0.05.

## Results

### Population-based demographics during the outbreak

**Figure 2:**
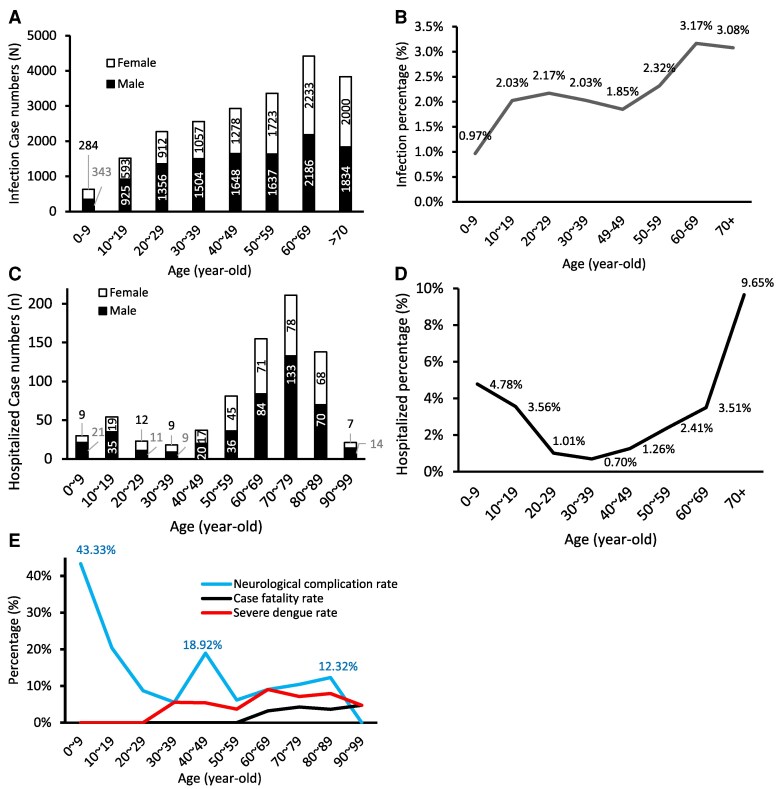
**Demographic characteristics and clinical outcomes of dengue patients.** (**A**) Age-stratified infection case numbers of symptomatic dengue patients across age intervals, stratified by sex. (**B**) Age-specific infection rates of symptomatic dengue patients. (**C**) Age-stratified case numbers of hospitalized dengue patients across age intervals, stratified by sex. (**D**) Age-specific hospitalization rates, calculated as the percentage of hospitalized patients relative to infected patients within each age group. (**E**) Age-specific proportions of hospitalized dengue patients presenting with neurological manifestations, severe dengue syndrome, and the case fatality rate (CFR). Bars in panels (**A**) and (**C**) represent male and female patients.

The study population and patient selection process are summarized in Fig. 1. Between January 1, 2023, and December 31, 2023, a total of 21 513 symptomatic dengue cases were reported to the NIRDS in Tainan. The largest number of cases occurred in individuals aged 60–69 years (Fig. 2A). After adjustment for age-specific population size, the highest infection rates were observed in individuals aged ≥70 years (Fig. 2B).

After excluding duplicate registrations (*n* = 12) and cases not ultimately diagnosed as dengue fever (*n* = 185), a total of 768 dengue patients were hospitalized in tertiary medical centres, including National Cheng Kung University Hospital (NCKUH; *n* = 365) and Chi Mei Medical Centre (CMH; *n* = 403), accounting for 3.6% of all reported dengue cases (Fig. 1). Paediatric patients (*n* = 78) constituted 10.2% of hospitalized cases. The mean age of adult hospitalized dengue patients was 67.5 ± 15.7 years. The age distribution of hospitalized patients demonstrated peaks in the 10–19 and 70–79 age groups (Fig. 2C). Hospitalization rates exhibited a bimodal pattern, with higher rates among patients younger than 19 years and those aged ≥70 years (Fig. 2D).

All cases of severe dengue syndrome occurred in adults, with a higher incidence among patients older than 60 years (Fig. 2E). A total of 20 patients (13 males) died from dengue infection, with a mean age of 76.1 ± 8.0 years, which was significantly older than that of hospitalized adult dengue patients (*P* = 0.015, *t*-test). The case fatality rate (CFR) was 2.6% among hospitalized patients and 0.9 per 1000 among all reported cases. Among the fatal cases, 17 were infected with dengue virus serotype 1, while the remaining three were associated with serotype 2.

### Neurological manifestations of dengue

Among all hospitalized dengue patients, 12% (*N* = 92) presented with neurological manifestations. Of these patients, 75% were adults (*N* = 69) and 25% were paediatric patients (*N* = 23). Neurological manifestations were observed in 10.0% of hospitalized adult patients and 29.5% of hospitalized paediatric patients, with a higher proportion observed in the paediatric population. Using all reported dengue cases in Tainan as the denominator, neurological manifestations accounted for 0.43% of the total disease burden.

Neurological manifestations were categorized as infection-related or immune-mediated parainfectious events.^7,10,15,23^ Infection-related manifestations included: (1) Direct or cytokine-mediated effects of acute infection, such as myositis (*N* = 60) and encephalitis with or without seizures (*N* = 3); (2) Secondary complications, including traumatic ICH associated with thrombocytopenia (*N* = 8) and metabolic encephalopathy due to hyponatremia or acute kidney injury (AKI) (*N* = 15); and (3) Exacerbation of pre-existing neurological disorders during acute infection, such as MG crisis or acute exacerbation (*N* = 2) and seizure recurrence in patients with epilepsy (*N* = 8). Immune-mediated parainfectious events included Guillain–Barré syndrome (GBS) (*N* = 6), myelitis (*N* = 1), new-onset MG (*N* = 1), and opsoclonus–myoclonus syndrome (*N* = 1).

#### Neurological manifestations in adult dengue patients

A comparison between adult dengue patients with and without neurological manifestations revealed no significant difference in age (*P* = 0.99, *t*-test). However, patients with neurological manifestations had a significantly higher proportion of males (*P* = 0.0009, Fisher's exact test), higher CCI scores (*P* = 0.0006, Mann–Whitney test), a greater prevalence of hypokalaemia (*P* = 0.0421, Fisher's exact test), and a lower prevalence of leukopoenia (*P* = 0.0022, Fisher's exact test; Supplementary Table 1).

Among all neurological manifestations, myositis was the most common neurological manifestation, accounting for 5.7% (*N* = 39) of hospitalized adult dengue patients and 56.5% of dengue patients with neurological manifestations, followed by encephalopathy, which occurred in 2.0% of hospitalized adult patients (*N* = 14). Higher CCI scores were observed in patients with myositis, encephalopathy, and ICH (Table 1).

**Table 1:** Characteristics of hospitalized adult dengue patients with neurological manifestation

	Myositis *N* = 39	Encephalopathy *N* = 14	ICH *N* = 8	Stroke *N* = 7	Seizure *N* = 7	GBS *N* = 6	MG *N* = 3	Encephalitis *N* = 3	Myelitis *N* = 1	Opsoclonus-myoclonus *N* = 1	All adult dengue patient *N* = 690
Proportion in hospitalized adult dengue (%)	5.7%	2.0%	1.2%	1.0%	1.0%	0.9%	0.4%	0.4%	0.1%	0.1%	100%
Age (mean ± SD)	65.5 ± 17.9	72.2 ± 12.5	75.8 ± 14.6	67.6 ± 12.5	54.0 ± 16.0*	69.3 ± 4.4	61.7 ± 14.7	66.3 ± 11.2	70	79	67.5 ± 15.7
Gender (Male, %)	74.4%*	57.1%	50%	85.7%	71.4%	83.3%	67%	33.3%	100%	100%	55.2%
CCI score(median, [IQR])	4, [1, 6]*	5, [2, 6.25]*	6, [2.25, 6]*	4, [1, 5]	4, [0, 6]	3, [1.5, 3.25]	4, [0, 9]	4, [4, 8]	3	3	2, [1, 4]
Expired (*n*, %)	6, 15.4%**	2, 14.3%	2, 25%*	0, 0%	0, 0%	0, 0%	0, 0%	1, 33.3%	0, 0%	0, 0%	20, 2.9%
Dengue features											
Leukopoenia (%)	35.9%*	35.7%	50%	28.6%	42.9%	0%**	33.3%	0%	0%	100%	55.2%
Thrombocytopenia (%)	82.1%	85.7%	75%	85.7%	85.7%	33.3%*	66.7%	100%	100%	100%	78.4%
Hyponatremia (<136 mEq) (%)	74.4%	64.3%	62.5%	71.4%	85.7%	83.3%	33.3%	100%	100%	100%	70.7%
Hypokalaemia (<3.6 mEq) (%)	66.7%*	78.6%*	62.5%	14.3%	71.4%	16.7%	33.3%	66.7%	100%	100%	47.5%
Hepatitis (%)	61.5%*	50%	37.5%	57.1%	71.4%	33.3%	66.7%	100%	0%	0%	42.3%

Statistics: All column compared with the ‘all adult dengue patient’. Age, *t*-test; sex, death, dengue features, Fisher’s exact test; CCI score, Mann–Whitney test.

CCI, Charlson comorbidity index; GBS, Guillain-Barre syndrome; ICH, intracranial haemorrhage; MG, myasthenia gravis.

^*^ *P* < 0.05.

^**^ *P* < 0.01.

#### Neurological manifestations in paediatric dengue patients

Among paediatric dengue patients, myositis was the most common neurological manifestation, accounting for 91.3% (*N* = 21) of children’s neurological manifestations. Myositis occurred in 26.9% of hospitalized paediatric patients, followed by seizures (4.3%) and encephalopathy (4.3%). No cases of GBS, myelitis, encephalitis, stroke, or ICH were reported in paediatric patients.

Clinical outcomes in paediatric patients were favourable, with no reported fatalities. No significant differences were observed in age, sex distribution, or dengue-related clinical characteristics between paediatric patients with and without neurological manifestations (Supplementary Table 1).

### Risk factors for developing neurological complications

Comparison between hospitalized dengue patients with and without neurological manifestations showed a higher proportion of males and a greater prevalence of hepatitis during acute dengue infection among patients with neurological manifestations (*P* = 0.0003 and *P* = 0.043, respectively; Fisher’s exact test). Among comorbidities, CKD and neurodegenerative diseases were associated with a higher likelihood of neurological manifestations (*P* = 0.033 and *P* = 0.037, respectively; Fisher’s exact test) (Table 2).

**Table 2:** Demographics, biological features, and risk factors of patients developing neurological manifestations of dengue

	With neurological manifestation (*N* = 92)	Without neurological manifestation (*N* = 676)	Univariate analysis		Multivariable analysis	
Variables			Crude OR (95% CI)	*P*-value	Adjusted OR (95% CI)	*P*-value
Male (*N*, %)	68, 73.9%***^,a^	365, 54.0%	2.41 (1.48–3.94)	<0.001***	2.20 (1.33–3.63)	0.002**
Age						
0∼19			Ref.		Ref	
20∼39			0.20 (0.06–0.70)	0.012*	0.15 (0.04–0.54)	0.004**
≥40			0.28 (0.16–0.48)	<0.001***	0.20 (0.11–0.36)	<0.001***
Features of dengue fever						
Leukopoenia	47, 51.1%	397, 58.7	0.73 (0.47–1.14)	0.165		
Thrombocytopenia	72, 78.3%	512, 75.7	1.15 (0.68–1.95)	0.595		
Hepatitis	47, 51.1%*^,a^	270, 40.1	1.57 (1.01–2.43)	0.043*	2.02 (1.26–3.23)	0.004**
Hyponatremia	58, 63.0%	462, 68.3	0.79 (0.50–1.24)	0.308		
Hypokalaemia	43, 46.7%	295, 43.6	1.13 (0.73–1.75)	0.574		
Comorbidities						
CCI scores	3, [0, 5]	2, [0, 4]	1.05 (0.97–1.13)	0.199		
Hypertension	40, 43.5%	312, 46.2%	0.90 (0.58–1.39)	0.629		
Diabetes mellitus	24, 26.1%	192, 28.4%	0.89 (0.54–1.45)	0.632		
Dyslipidemia	19, 20.7%	171, 25.3%	0.76 (0.45–1.3)	0.323		
Hematologic disease	2, 2.2%	20, 3.0%	0.73 (0.17–3.17)	0.672		
Cancer	13, 14.1%	111, 16.4%	0.83 (0.45–1.54)	0.549		
Heart disease	24, 26.1%	140, 20.7%	1.35 (0.82–2.23)	0.242		
COPD or asthma	8, 8.7%	37, 5.5%	1.64 (0.74–3.65)	0.223		
Liver cirrhosis	2, 2.2%	13, 1.9%	1.13 (0.25–5.1)	0.872		
Autoimmune disease	7, 7.6%	32, 4.7%	1.65 (0.71–3.87)	0.245		
CVA	7, 7.6%	70, 10.4%	0.71 (0.32–1.6)	0.411		
CKD	23, 25.0%*^,a^	108, 16.0%	1.75 (1.05–2.93)	0.033*	2.31 (1.32–4.07)	0.004**
Genetic disease	1, 1.1%	4, 0.6%	1.84 (0.20–16.67)	0.586		
Neurodegenerative disease	12, 13.0%*^,a^	46, 6.8%	2.05 (1.04–4.03)	0.037*	2.81 (1.36–5.84)	0.005**
Psychiatric disease	4, 4.3%	12, 1.8%	2.51 (0.79–7.96)	0.118		

CCI, Charlson comorbidity Index; CKD, Chronic kidney disease; COPD, Chronic obstructive pulmonary disease; CVA, Cerebrovascular accident.

^a^Chi-square test.

^*^ *P* < 0.05.

^**^ *P* < 0.01.

^***^ *P* < 0.001.

Univariate analysis followed by multivariable logistic regression identified male sex, concurrent hepatitis, comorbid CKD, and neurodegenerative disease as independent risk factors for neurological manifestations in hospitalized dengue patients (Table 2). Male sex was associated with a 2.20-fold increased risk of neurological complications (OR 2.20, 95% CI 1.33–3.63). Similarly, hepatitis, CKD, and neurodegenerative disease were associated with increased risks, with odds ratios of 2.02 (95% CI 1.26–3.23), 2.31 (95% CI 1.32–4.07), and 2.81 (95% CI 1.36–5.84), respectively (Table 2).

### Male predominance in symptomatic dengue, severe dengue, and dengue with neurological manifestations

**Figure 3:**
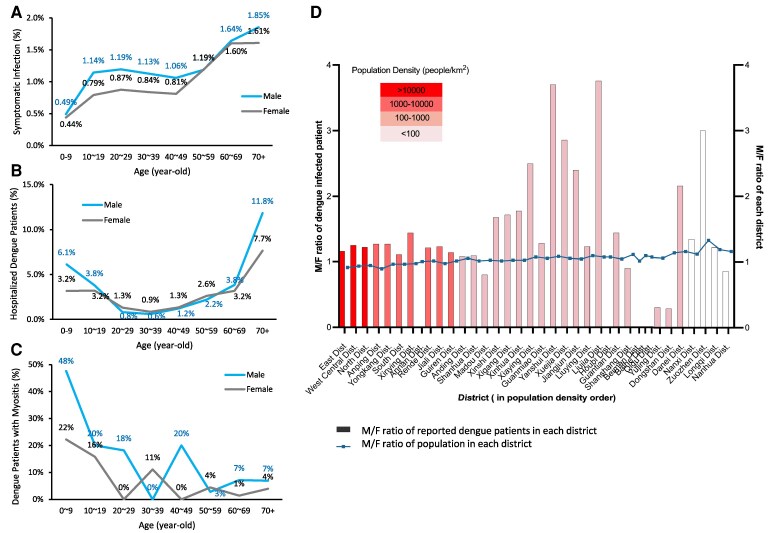
**Male predominance across dengue infection, hospitalization, and myositis.** (**A–C**) Age- and sex-specific distributions of dengue patients across age intervals, including (**A**) symptomatic infection, (**B**) hospitalization, and (**C**) dengue patients with myositis. Male and female patients are shown separately for each age group. (**D**) Male-to-female ratio of reported symptomatic dengue infections across 37 districts in Tainan. Bars represent the male-to-female ratio of dengue cases in each district (left Y-axis) and are grouped according to population density (people/km²). The male-to-female ratio of the population in each district is shown as a dotted line and referenced to the right Y-axis. Linear regression analysis between district population and dengue case male-to-female ratios demonstrated a weak correlation (*R*^2^ = 0.064, Pearson correlation). Districts without reported dengue cases were excluded from the correlation analysis.

An age- and sex-stratified analysis demonstrated a consistent male predominance across multiple stages of dengue infection, including symptomatic infection, hospitalization, and dengue-associated neurological complications (Fig. 3). As shown in Fig. 3A–C, higher proportions of male patients were observed across most age groups for symptomatic dengue, hospitalization, and dengue-related myositis. Age-specific male and female proportions are provided in Supplementary Fig. 1.

#### Symptomatic dengue

During the 2023 Tainan dengue outbreak, symptomatic dengue infection demonstrated a clear male predominance. Overall, males accounted for 53% of all registered symptomatic cases and had a modest but statistically significant increased risk compared with females (crude OR 1.15, 95% CI 1.12–1.18, Table 3 and Supplementary Fig. 2A).

**Table 3:** Odds ratio of infection rate, hospitalization rate, and neurological manifestation rate in various age groups

	Infected (*N* = 21 513)		Hospitalized (*N* = 768)		Neurological manifestations (*N* = 92)				
Age	Crude OR (95 CI)	*P* value	Crude OR (95 CI)	*P* value	Crude OR (95 CI)	*P* value	Adjusted OR (95 CI)	*P* value	*P* for trend
0–19	1.34 [1.23–1.47]	<0.001***	1.41 [0.90–2.21]	0.1743	1.74 [0.61–5.14]	0.4427	1.65 [0.56–4.89]^a^	0.366	0.043*
20–39	1.36 [1.28–1.44]	<0.0001****	0.65 [0.35–1.20]	0.2017	2.22 [0.23–33.60]	0.6060	1.76 [0.23–13.53]^a^	0.588	
>40	1.09 [1.06–1.13]	<0.0001****	1.25 [1.06–1.46]	0.0068**	2.46 [1.37–4.39]	0.0016**	2.37 [1.32–4.26]^a^	0.004**	
All	1.15 [1.12–1.18]	<0.0001****	1.15 [0.99–1.32]	0.0673	2.41 [1.49–3.94]	0.0003***	2.21 [1.34–3.66]	0.002**	

^a^Adjustment was made for comorbidity.

^*^ *P* < 0.05.

^**^ *P* < 0.01.

^***^ *P* < 0.001.

^****^ *P* < 0.0001.

Age-stratified analyses showed that this male predominance was consistently observed across most age groups (Fig. 3A). The proportion of male cases was particularly higher among individuals younger than 50 years (Supplementary Fig. 1A), in whom males comprised 58.34% of symptomatic dengue cases.

Population-based analyses further supported the observed male predominance. Normalization of the male-to-female (M/F) ratio of symptomatic dengue cases against the underlying population M/F ratio across Tainan’s 37 districts showed that, after excluding five districts with no reported cases, 26 of the remaining 32 districts (81.3%) exhibited an M/F infection ratio >1, with a weak correlation between population M/F ratio and infection M/F ratio (*R*^2^ = 0.0644; Fig. 3D).

A male-predominant pattern was observed across districts with varying population densities (Fig. 3D). In highly urbanized districts (>10 000 people/km^2^, red bars in Fig. 3D), the M/F infection ratio ranged from 1 to 1.5, despite population M/F ratios below 1 in most districts. In lower-density districts (<1000 people/km^2^, light pink and white bars in Fig. 3D), the M/F infection ratio exceeded 1 in 18 of 22 districts and showed greater variability (range, 1–4), likely reflecting smaller numbers of reported cases.

#### Hospitalized dengue

Among dengue patients admitted to tertiary medical centres, males constituted a greater proportion of hospitalized cases, accounting for 56.4% of all admissions, including 55.2% of adult patients and 66.7% of paediatric patients. At the population level, however, male sex was not associated with a significantly increased overall risk of hospitalization compared with females (OR 1.15, 95% CI 0.99–1.32; Table 3). Consistent with this finding, overall hospitalization rates were similar between sexes, at 3.8% in males and 3.3% in females (*P* = 0.067, chi-square test).

Age-stratified analyses revealed clear variation in hospitalization patterns across age groups (Fig. 3B). Male hospitalization rates tended to be higher at younger and older ages, with the greatest separation between sexes observed among patients aged ≥70 years, whereas the differences were less pronounced in middle-aged groups. In terms of sex composition, males constituted a larger proportion of hospitalized patients across most age intervals, with male-to-female ratios exceeding 1 in nearly all age groups (Supplementary Fig. 1B).

Further stratified analyses demonstrated that sex differences in hospitalization risk were age dependent. After adjustment for potential confounders, males aged >40 years had a significantly higher risk of hospitalization compared with females (crude OR 1.25, 95% CI 1.06–1.46; Table 3 and Supplementary Fig. 2B). Male predominance was also observed among fatal cases, with males accounting for 65% of the 20 dengue-related deaths.

#### Dengue with neurological menifestations

Males were more likely to develop neurological complications among hospitalized dengue patients, with a 2.2-fold increased risk after adjustment for comorbidities (crude OR 2.41, 95% CI 1.49–3.94, *P* = 0.0003; adjusted OR 2.21, 95% CI 1.34–3.66, *P* = 0.002, logistic regression; Table 3). Given the potential modifying effect of age on dengue severity, we further examined whether this male predominance varied across age groups. Age-stratified analyses showed that the increased risk associated with male sex was particularly pronounced in patients aged >40 years (crude OR 2.46, 95% CI 1.37–4.39, *P* = 0.0016; adjusted OR 2.37, 95% CI 1.32–4.26, *P* = 0.004, logistic regression; Table 3 and Supplementary Fig. 2C). Trend analysis further demonstrated that the risk of neurological complications in males increased with age (*P* = 0.043, Table 3).

When individual neurological manifestations were examined, a male predominance was observed across most conditions in adults, with the exception of encephalitis and ICH, in which males accounted for 33.3% and 50% of cases, respectively (Table 1). In contrast, no clear sex-associated differences were observed among paediatric patients with neurological manifestations.

Among the various neurological complications, myositis showed the most consistent male predominance in adults. In adult patients with dengue-related myositis, 74.4% were male, which was significantly higher than the proportion of males in the overall adult dengue population (55.2%, *P* = 0.0202, Fisher’s exact test; Table 1). Age-stratified analysis of case distribution further demonstrated that males constituted a larger proportion of myositis cases across most age groups, although variability was noted in strata with small case numbers (Supplementary Fig. 1C).

To determine whether this male predominance could be explained by differences in hospitalization structure across age groups, sex-specific myositis rates were further analyzed using the number of hospitalized male and female dengue patients in each age interval as the denominator. After this adjustment, males consistently exhibited higher myositis rates than females across most age groups (Fig. 3C). Although variability was observed in middle-aged strata, particularly the 30–39 and 50–59 age intervals where case numbers were limited (one and three cases, respectively), male predominance was observed across multiple age groups rather than being confined to a single age interval.

### Clinical subtype of dengue neurological manifestations

Neurological manifestations observed in this cohort were classified according to their temporal relationship to acute dengue infection. Acute infection–related neurological complications included manifestations attributable to direct viral involvement, cytokine-mediated processes, or systemic complications, such as myositis, encephalitis and encephalopathy, ICH, seizures, and MG crisis. Parainfectious immune-mediated syndromes comprised GBS, new-onset MG, and opsoclonus–myoclonus syndrome. Several subtypes, including encephalitis, myelitis, MG, and opsoclonus–myoclonus syndrome, were infrequently observed, and analyses involving these manifestations were therefore limited by small case numbers.

#### Acute infection-related neurological complications

##### Dengue-related myositis

Myositis was the most common neurological manifestation among dengue patients hospitalized in tertiary medical centres, affecting 5.7% of adults and 26.9% of paediatric patients. The age distribution demonstrated a bimodal pattern, with peaks in children aged 0–9 years and older adults aged 70–79 years (Supplementary Fig. 3A). Patients younger than 20 years had a significantly higher prevalence of myositis than those aged ≥20 years (26.2% versus 5.6%, *P* < 0.0001, Fisher’s exact test; Supplementary Fig. 3B).

Among adult dengue patients, those with myositis had higher comorbidity burdens, reflected by higher CCI scores [median (IQR), 4 (1–6) versus 2 (1–4), *P* = 0.0221]. Compared with the overall hospitalized adult dengue population, adult patients with myositis showed a lower prevalence of leukopoenia (35.9% versus 55.2%, *P* = 0.0208), and higher prevalences of hypokalaemia (66.7% versus 47.5%, *P* = 0.0215) and hepatitis (61.5% versus 42.3%, *P* = 0.0204). Clinical outcomes were poorer in adults with myositis, with a higher CFR than in those without myositis (15.4% versus 2.9%, *P* = 0.0017; Table 1).

In contrast, laboratory profiles among paediatric dengue patients with myositis did not differ significantly from those without myositis (Supplementary Table 2).

##### Other acute infection–related neurological manifestations

Dengue encephalitis was diagnosed in three patients, two of whom experienced prolonged hospital stays, while one elderly patient died early during hospitalization. Encephalopathy observed in other patients was primarily associated with metabolic derangements and was accompanied by higher CCI scores (*P* = 0.0156) and a higher prevalence of hypokalaemia (*P* = 0.0286) compared with the overall hospitalized cohort (Table 1).

All ICH cases were associated with head trauma. The prevalence of thrombocytopenia among patients with ICH (75%) was comparable to that of the overall hospitalized cohort (78.4%, *P* = 0.6853). Patients with ICH had higher comorbidity burdens and a higher CFR (25%, *P* = 0.0237; Table 1). Seven patients developed ischaemic stroke within 2 months after dengue infection; most had pre-existing vascular risk factors, and a causal relationship with dengue infection could not be definitively established.

Seizures occurred at a median of 4.5 days after symptom onset [median (IQR), 4.5 (2.5–6.75) days]. Most episodes were febrile-provoked seizures in patients with pre-existing epilepsy, whereas a minority represented new-onset seizures associated with encephalitis.

Exacerbation of MG occurred shortly after dengue onset in patients with both acetylcholine receptor antibody–positive and anti–MuSK antibody–positive disease. In the absence of early inflammatory control, progression to myasthenic crisis was observed at a median of 8.5 days after symptom onset. All affected patients received plasma exchange–based therapy and achieved favourable recovery.

#### Immune-mediated parainfectious neurological complications

GBSeloped at a median of 5 days after fever onset. At the time of neurological evaluation, dengue-related leukopoenia and thrombocytopenia had largely resolved, and patients with GBS showed lower prevalences of both abnormalities compared with the overall hospitalized cohort (*P* = 0.0068 and *P* = 0.0233, respectively; Table 1). Most patients underwent plasma exchange, and clinical improvement was observed in all cases.

A single case of opsoclonus–myoclonus syndrome was identified in an elderly patient without typical dengue symptoms. Severe thrombocytopenia and leukopoenia were present, while brain MRI was unremarkable. Neurological symptoms resolved completely within 20 days.

**Figure 4:**
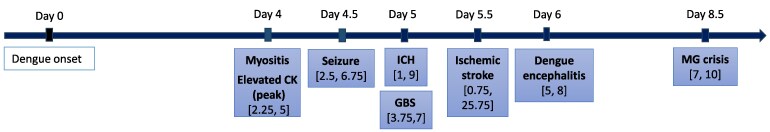
**Timeline of neurological manifestations in dengue infection.** Timeline illustrating the onset of neurological manifestations among hospitalized dengue patients. The onset day is defined as the day of the first fever peak. Numbers above the timeline indicate the median onset day for each neurological manifestation, with parentheses showing the interquartile range (first and third quartiles). CK, creatinine kinase; ICH, intracranial haemorrhage; GBS, Guillain Barre syndrome; MG, myasthenia gravis.

The median onset timing of each neurological manifestation during the course of dengue infection is summarized in Fig. 4.

## Discussion

This population- and hospital-based study provides a comprehensive characterization of sex differences in dengue-associated neurological complications during a large community outbreak. We demonstrate a consistent male predominance across multiple stages of disease expression, including symptomatic infection, hospitalization, severe dengue, and neurological involvement, indicating that sex is an important determinant of dengue susceptibility and complication risk.

Myositis was the most common neurological manifestation in both adults and children. Among adults, myositis and encephalopathy accounted for 5.7% and 2.0% of hospitalized cases, respectively, whereas in paediatric patients, myositis predominated, affecting more than one quarter of hospitalized children. In parallel, males exhibited a higher risk of symptomatic dengue infection across the entire population (OR 1.153), and males aged >40 years were more likely to require hospitalization and to develop neurological complications during admission.

Male predominance in dengue infection and disease severity has been reported previously.^15^ Although some epidemiological studies have attributed this disparity to social or gender-related factors,^16,17^ such explanations are less plausible in Taiwan, where access to healthcare is universal. Moreover, the excess of symptomatic dengue observed in young children, an age group in which differential exposure is unlikely, supports a biological basis for male susceptibility.

Male disadvantage in infectious disease severity is not unique to dengue but has also been described in other viral infections, including poliomyelitis,^26^ syncytial respiratory virus,^27^ SARS-CoV-2,^28-31^ and bacterial infections like meningococcal meningitis.^32^ MS Creen proposed a conceptual framework suggesting that relative immunodeficiency in males, encompassing a spectrum of impairments in humoral and cellular immune responses, may underlie this phenomenon.^33^ Consistent with this model, males generally exhibit weaker innate and adaptive immune responses than females, whereas females mount more robust immune reactions that facilitate pathogen clearance and vaccine responses but also confer increased susceptibility to autoimmune and inflammatory diseases.^34,35^ These sex-based immunological differences, shaped by genetic and hormonal factors, may contribute to differential vulnerability to dengue infection and its complications.

In hyperendemic regions, repeated dengue exposure, cumulative immune priming, and antibody-dependent enhancement may substantially modify host immune responses, potentially obscuring intrinsic susceptibility factors at the population level. In contrast, dengue epidemiology in Taiwan is characterized by intermittent outbreaks separated by prolonged inter-epidemic periods, resulting in relatively limited prior population immunity. This epidemiological context provides a valuable opportunity to examine biological susceptibility with reduced confounding from repeated exposure. Accordingly, the consistent male predominance observed across symptomatic infection, hospitalization, and neurological complications in our cohort is more likely to reflect underlying sex-related biological vulnerability rather than differences driven primarily by cumulative immune imprinting or exposure patterns.

Cases included in this study were highly representative of the 2023 dengue outbreak in Tainan. The two participating healthcare systems—National Cheng Kung University Hospital, comprising one medical centre, two regional hospitals, and one district hospital, and the Chi Mei Medical Centre system, comprising one medical centre, one regional hospital, and one district hospital—together provide near-complete tertiary care coverage in the Tainan area. Consequently, the majority of patients with severe dengue and dengue-associated neurological manifestations were referred to the tertiary medical centres within these systems. Nevertheless, several limitations should be acknowledged. First, as a retrospective observational study, missing data may have led to underestimation of incidence. Second, the timing of dengue symptom onset was based partly on patient self-report and may therefore lack precision. Third, patients with mild or asymptomatic infection were not captured by the national reporting system. In addition, several neurological subgroups were rare, resulting in limited statistical power; accordingly, statistical findings for these entities should be interpreted as exploratory, with greater emphasis placed on absolute case numbers and effect sizes. Future prospective studies with standardized neurological assessments are warranted to refine epidemiological estimates and improve risk stratification during dengue outbreaks.

In summary, this study included all severe dengue patients and those with neurological manifestations during the 2023 outbreak. We identified a male-predominant incidence of symptomatic, severe, and complicated dengue. Additionally, we described the demographics and clinical presentations of neurological manifestations in dengue fever, which will help raise awareness among physicians and neurologists to recognize complications early and initiate proactive management. This will better prepare us for future endemic outbreaks, which are likely to recur.

## Supplementary Material

fcag258_Supplementary_Data

## Data Availability

Data are available upon reasonable request to the corresponding author.
