# Supplementary material for: Sex differences in dengue-related neurological complications: insights from the 2023 Taiwan outbreak
Source: Brain Commun. 2026 Jul 7;8(4):fcag258. doi: 10.1093/braincomms/fcag258 (PMC13378165; doi:10.1093/braincomms/fcag258)
Supplement: fcag258_Supplementary_Data [file fcag258_supplementary_data.pdf]

Supplementary Table 1 The demographic data and the features of dengue in patients with and without neurological manifestations

Demographics and features of dengue	**ADULTS (N=690)**		**CHILDREN (N=78)**	
	WITH	WITHOUT	WITH	WITHOUT
Demographics				
Case (N, %)	69, 10%	621, 90%	23, 29.5%	55, 70.5%
Gender (Male, %)	51, 73.9%***	330, 53.1%	17, 73.9%	35, 63.6%
Age (mean ± SD)	67.5 ± 16.2	67.5 ± 15.7	10.0 ± 4.0	10.5 ± 4.7
CCI score (median, [IQR])	4, [1.5,6]***	2, [1,4]	0, [0.0]	0, [0,0]
Biological features of dengue				
Leukopenia (N, %)	26, 37.7%**	355, 57.2%	21, 91.3%	42, 76.4%
Thrombocytopenia (N, %)	56, 81.2%	485, 78.1%	16, 69.6%	27, 49.1%
Hyponatremia (<136 mEq) (N, %)	49, 71.0%	439, 70.7%	9, 39.1%	23, 41.8%
Hypokalemia (<3.6 mEq) (N, %)	41, 59.4%*	287, 46.2%	2, 8.7%	8, 14.6%
Hepatitis (N, %)	36, 52.2%	256, 41.2%	11, 47.8%	15, 27.3%

CCI, Charlson comorbidity Index; *, p<.05; **, p<.01; ***, p<.001;

Statistics: Age, t-test; sex, dengue features, Fisher’s exact test; CCI score, Mann-Whitney test.

Supplementary Table 2 Characteristics of hospitalized pediatric dengue patients with myositis.

	Myositis<br>N=21	All pediatric dengue patient<br>N=78
Proportion in hospitalized pediatric dengue (%)	26.9%	100%
Age (mean ± SD)	10.0±4.1	10.4±4.6
Gender (Male, %)	76.2%*	66.7%
CCI score (median, [IQR])	0	0
Expired (n, %)	0, 0.0%	0, 0.0%
Dengue features		
Leukopenia (%)	95.2%	80.8%
Thrombocytopenia (%)	71.4%	55.1%
Hyponatremia (<136 mEq) (%)	33.3%	41.0%
Hypokalemia (<3.6 mEq) (%)	4.7%	12.8%
Hepatitis (%)	52.4%	32.1%

CCI, Charlson comorbidity index; *, p<.05; **, p<.01; ***, p<.001; ****, p<0.0001.

Statistics: Age, t-test; sex, death, dengue features, Fisher's exact test; CCI score, Mann-Whitney test.

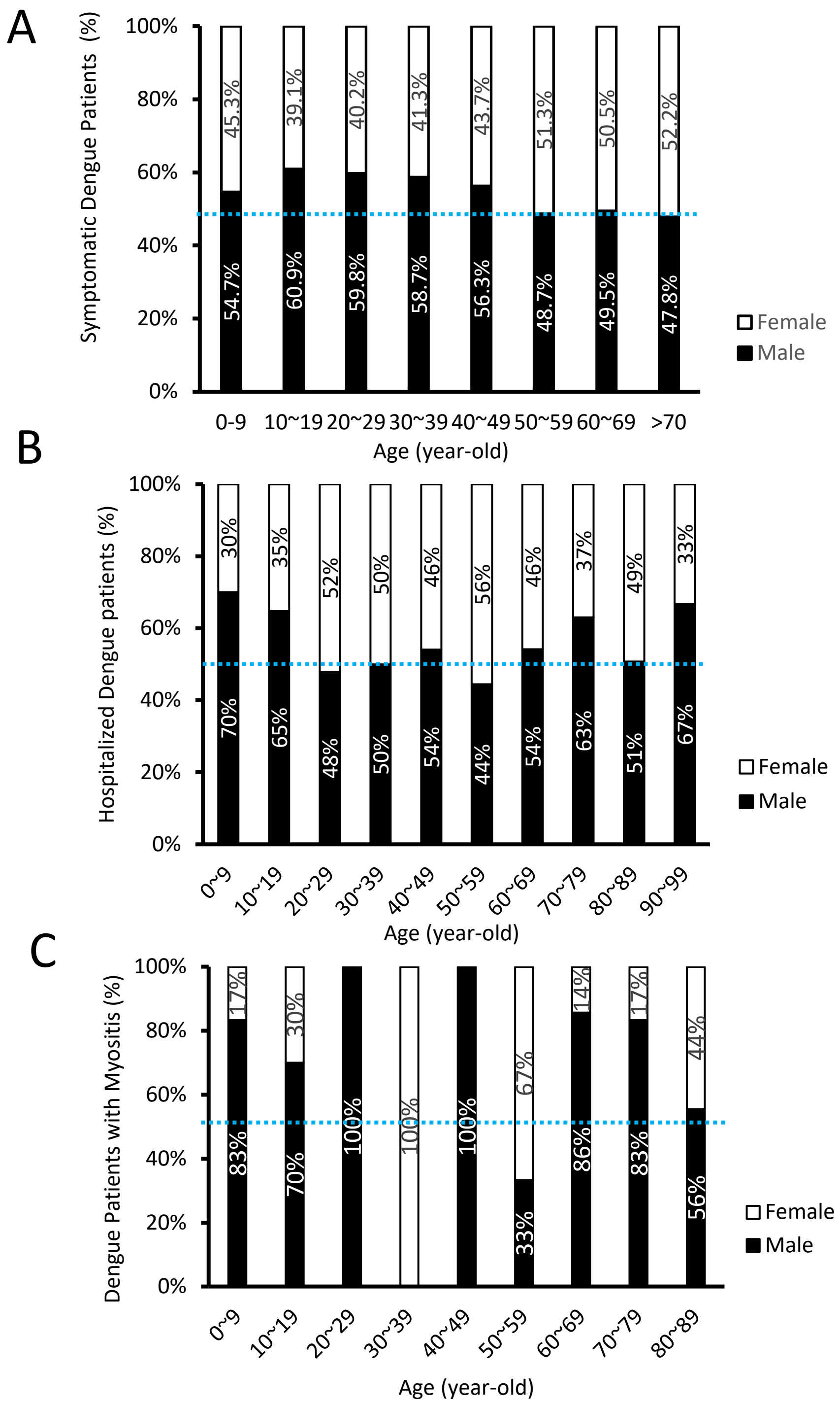

Supplementary Figure 1. Age-Specific Male and Female Distribution of Dengue Patients. (A–C) Age-specific male and female distributions across age intervals in **(A)** symptomatic dengue patients, **(B)** hospitalized dengue patients, and **(C)** dengue patients with myositis. Stacked bars show the percentage of male and female patients in each age group, with the dashed line indicating a 50% distribution.

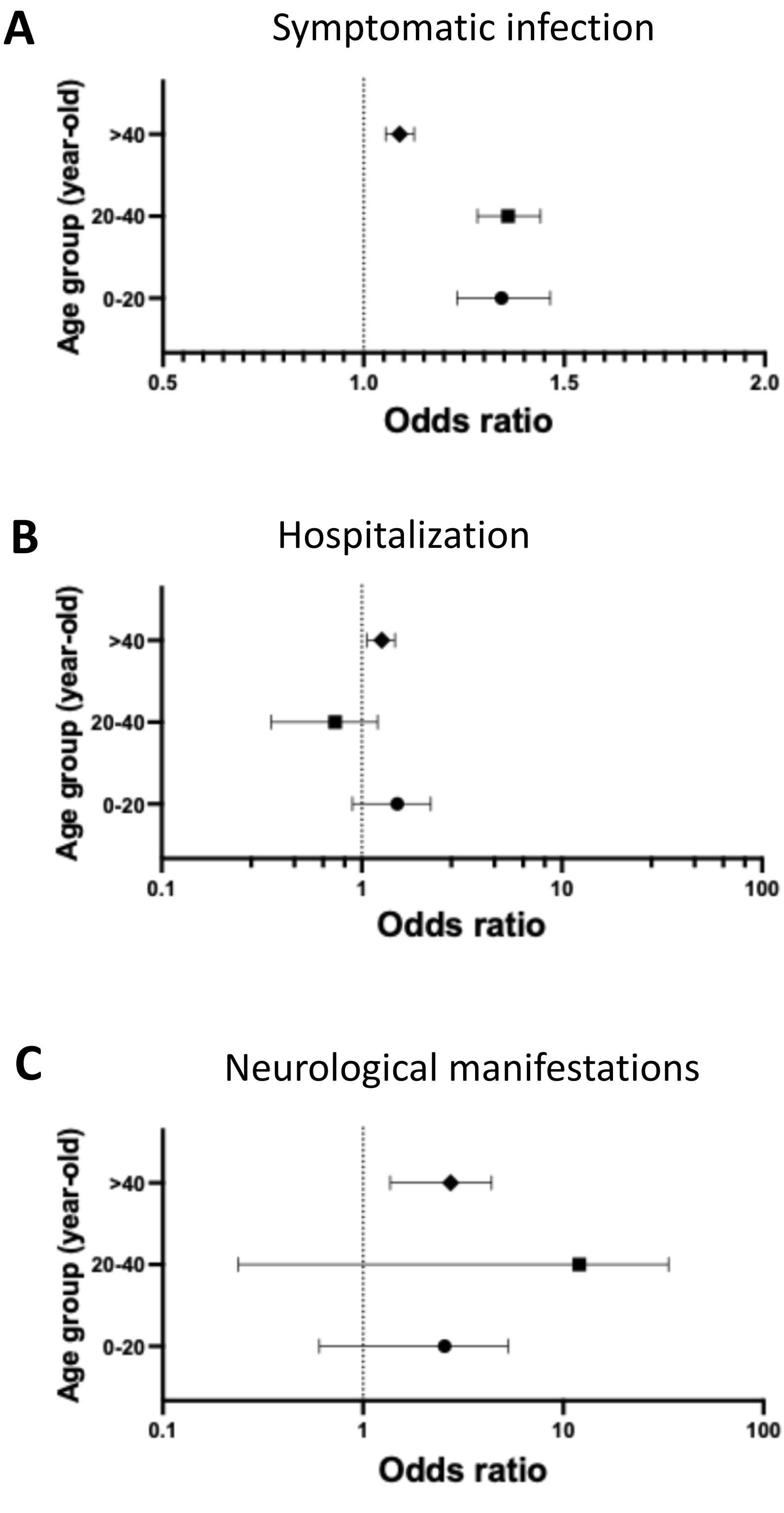

Supplementary Figure 2. Risk of Symptomatic Infection, Hospitalization, and Neurological Manifestations in Males Across Age-Stratified Groups. **(A)** Odds ratios (ORs) for males associated with symptomatic dengue infection across age groups (0–20, 20–40, and >40 years). Males accounted for 53% of all registered symptomatic cases and had a modest but statistically significant increased risk compared with females (crude OR 1.15, 95% CI 1.12–1.18). **(B)** ORs for males associated with hospitalization among symptomatic dengue patients across the same age groups. Males aged >40 years had a significantly higher risk of hospitalization compared with females (crude OR 1.25, 95% CI 1.06–1.46). **(C)** ORs for males associated with neurological manifestations among hospitalized dengue patients across the same age groups. Data are presented as OR with 95% confidence intervals. The dotted vertical line indicates an OR of 1. Male sex was particularly pronounced in patients aged >40 years (crude OR 2.46, 95% CI 1.37–4.39, P = 0.0016; adjusted OR 2.37, 95% CI 1.32–4.26, P = 0.004, logistic regression)

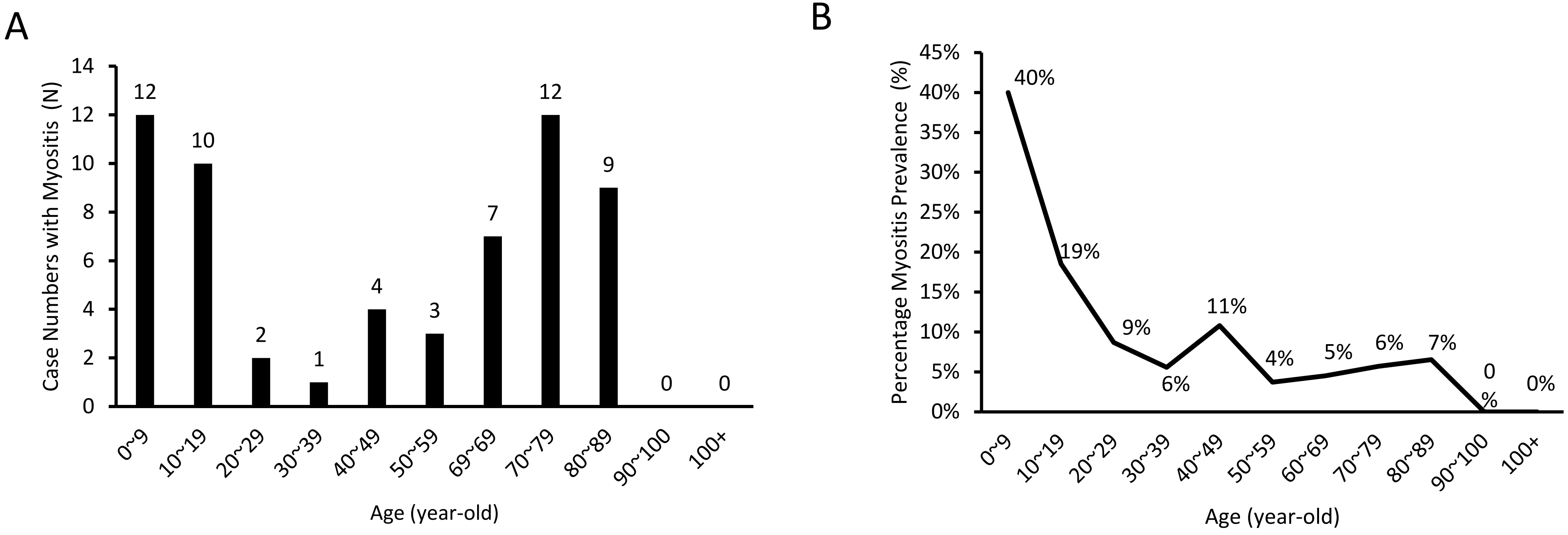

Supplementary Figure 3. Age-Related Distribution of Myositis Among Hospitalized Dengue Patient. (A, B) Case numbers **(A)** and age-specific prevalence **(B)** of myositis among hospitalized dengue patients across age intervals. Prevalence was calculated as the number of myositis cases in each age group divided by the total number of hospitalized dengue patients within the same age group. Patients younger than 20 years had a significantly higher prevalence of myositis than those aged ≥20 years (26.2% vs. 5.6%, $P < 0.0001$, Fisher's exact test)
